# HIV prevention costs and their predictors: evidence from the ORPHEA Project in Kenya

**DOI:** 10.1093/heapol/czx121

**Published:** 2017-09-26

**Authors:** Omar Galárraga, Richard G Wamai, Sandra G Sosa-Rubí, Mercy G Mugo, David Contreras-Loya, Sergio Bautista-Arredondo, Helen Nyakundi, Joseph K Wang’ombe

**Affiliations:** 1 School of Public Health, Brown University, Providence, RI, USA,; 2 Global Health Initiative, Northeastern University, Boston, MA, USA,; 3 Health Economics Unit, Mexican Institute of Public Health, Cuernavaca, Mexico,; 4 Department of Economics, University of Nairobi, Nairobi, Kenya,; 5 School of Public Health, University of California at Berkeley, Berkeley, CA, USA and; 6 School of Public Health, University of Nairobi, Nairobi, Kenya

**Keywords:** HIV/AIDS prevention, costs, HIV testing and counselling, prevention of mother-to-child transmission, voluntary medical male circumcision, economics

## Abstract

We estimate costs and their predictors for three HIV prevention interventions in Kenya: HIV testing and counselling (HTC), prevention of mother-to-child transmission (PMTCT) and voluntary medical male circumcision (VMMC). As part of the ‘*Optimizing the Response of Prevention: HIV Efficiency in Africa*’ (*ORPHEA*) project, we collected retrospective data from government and non-governmental health facilities for 2011–12. We used multi-stage sampling to determine a sample of health facilities by type, ownership, size and interventions offered totalling 144 sites in 78 health facilities in 33 districts across Kenya. Data sources included key informants, registers and time-motion observation methods. Total costs of production were computed using both quantity and unit price of each input. Average cost was estimated by dividing total cost per intervention by number of clients accessing the intervention. Multivariate regression methods were used to analyse predictors of log-transformed average costs. Average costs were $7 and $79 per HTC and PMTCT client tested, respectively; and $66 per VMMC procedure. Results show evidence of economies of scale for PMTCT and VMMC: increasing the number of clients per year by 100% was associated with cost reductions of 50% for PMTCT, and 45% for VMMC. Task shifting was associated with reduced costs for both PMTCT (59%) and VMMC (54%). Costs in hospitals were higher for PMTCT (56%) in comparison to non-hospitals. Facilities that performed testing based on risk factors as opposed to universal screening had higher HTC average costs (79%). Lower VMMC costs were associated with availability of male reproductive health services (59%) and presence of community advisory board (52%). Aside from increasing production scale, HIV prevention costs may be contained by using task shifting, non-hospital sites, service integration and community supervision.

Key Messages

- In Kenya, during 2011–12, the costs per client in HIV testing and counselling (HTC) was $7, for prevention of mother-to-child transmission (PMTCT) client tested was $59 and per voluntary medical male circumcision (VMMC) procedure was $66.
- Economies of scale were observed for the production of HIV prevention services particularly for PMTCT and for VMMC.
- Task shifting was associated with lower costs for PMTCT and for VMMC, but not for HTC.
- Incentives for good performance for staff members were not associated with higher costs.
- The availability of male reproductive health services and the presence of a community advisory board were associated with lower VMMC costs.

## Introduction

HIV is a serious public health problem in Kenya with 1.6 million people including 191 000 children infected (5.6% prevalence) (NACC and NASCOP 2014). In 2013 over 50 000 new HIV infections were documented among women, over 38 000 among men, and ∼13 000 among children (NACC and NASCOP 2014). The distribution shows remarkable geographical diversity with Homa Bay county in Lake Victoria region having the highest prevalence of 25.7% whereas Wajir County in north-eastern region had the lowest at 0.2% (NACC and NASCOP 2014), raising the importance of local population targeting with the most cost-effective interventions to maximize the effects of HIV prevention and treatment (Anderson *et al.* 2014; UNAIDS 2015b; Cassels and Camlin 2016; Chang *et al.* 2016; Coburn *et al.* 2017).

Kenya was a signatory country of the 2011 United Nations (UN) Political Declaration on HIV and AIDS (General Assembly Resolution No. 65/277) (universal access targets), adopted in June 2011 at the UN General Assembly High‐Level Meeting on AIDS, and the country has now moved towards implementing the Sustainable Development Goals (SDGs) (UN 2017). The new targets include reduced annual new HIV infections among adults by 75%, and reduced HIV transmission rates from mother to child from 14 to < 5% (NACC 2016a). Significant progress has been made in stemming the tide in Kenya (Kimanga *et al.* 2014), such as the reduction of national prevalence from a peak of ∼10% in the mid-1990s to 5.6% in 2014, and annual incidence from nearly 300 000 to 100 000 during the same period (NACC and NASCOP 2014; NASCOP 2014); however, a number of key national targets have not been met in part due to funding gaps (NACC 2014a). To reach the universal access targets for prevention and treatment set out in the *UNAIDS Getting to Zero 2011–2015 Strategy* (UNAIDS 2010), the country’s third National AIDS Strategic Plan (KNASP III, 2009/10–2012/13) estimated total resource requirement at $3.5 billion (58% treatment and care and 20% prevention) over the period, with a gap of nearly half of that ($1.7 billion) (NACC 2009). Similarly, estimates for treatment and prevention of mother-to-child transmission (PMTCT) alone for 2010–14 showed a funding gap of $1.8 billion (Government of the Republic of Kenya 2011). Furthermore, during 2010–20 the cost of the HIV response is estimated to increase by 114% with an overall funding gap of $1.75 billion or 0.3% of gross national product (GDP) by 2020 (UNAIDS 2013b). The latest published (2009/10–2011/12) National AIDS Spending Assessment (NASA) indicates that 62% of HIV expenditure was financed by donors (Republic of Kenya 2014). Overall expenditure decreased from $826 in 2009–10 to $786 million in 2011–12 (Republic of Kenya 2014) due in part to reduced PEPFAR bilateral dollars (PEPFAR 2011). A review of the KNASP III highlights the outstanding and future critical gaps in financing due to a real concern of withdrawal or termination of various donor funding agreements (NACC 2014b; NACC 2016c). Hence, in order to scale-up HIV services to achieve nationally set objectives and targets within a sustainability financing mechanism, it is paramount to optimize efficiency in resources use by HIV programs.

Like other high prevalence countries in sub-Saharan Africa, achieving an AIDS-free generation in Kenya requires aggressive programming in stopping transmission by preventing new infections (UNAIDS 2010, 2014; Goosby *et al.* 2012; Wamai 2014). In Kenya, however, prevention programs receive <20% of the HIV budget (Republic of Kenya 2014; NACC 2016c); thus the need to focus on a few key priority pillars with proven effectiveness. One of these is expanding HIV testing and counselling (HTC) to increase the number of people aware of their status, given that still less than half (47%) of women and about a third of men (35.8%) have received a test in the past year and know their results (NACC 2014a). In addition, to achieve virtual elimination of mother-to-child transmission (Mahy *et al.* 2010), the country needs to urgently improve its program for PMTCT: its coverage slipped from 86% in 2010 to about 70% in 2013 due to increased demand (NACC 2014a), and it varies widely across the counties (NACC 2016b). A third pillar is the full implementation of the on-going policy program for voluntary medical male circumcision (VMMC), which although has reached initial targets of adult men, needs to reach younger men and infant boys (Bailey *et al.* 2007; NASCOP 2008; Mwandi *et al.* 2011; Galbraith *et al.* 2014; WHO 2016).

Given the continued need for expanded services, while resources are diminishing, the main objectives of this paper are to document the costs of HIV prevention interventions, explore the predictors of economic efficiency, and quantify the potential economies of scale in the production of HIV prevention services. We estimate average costs at each step of the service cascade for each intervention, and then quantify the relationship between average unit cost and scale of production (i.e. the number of clients) as well as quality indicators for each type of facility. We define economic (technical) efficiency as delivering a given level of HIV services output at the lowest feasible cost (Bautista-Arredondo *et al.* 2008; Bertozzi *et al.* 2008) while holding other characteristics constant, including quality. Under this framework, economies of scale imply a reduction in the average cost of services as the number of clients scales-up (see Supplementary Technical Appendix, Section 1, for additional details on defining and measuring efficiency). The existence of economies of scale in production has been theoretically and empirically associated with decreased costs (Over 1986; Dandona *et al.* 2005; Guinness *et al.* 2005; Boily *et al.* 2007; Marseille *et al.* 2007; Chandrashekar *et al.* 2010; Marseille *et al.* 2012). Our study also includes questions relevant to evaluate costs and efficiency determinants as identified in the literature (Preyra and Pink 2001; Kasymova *et al.* 2009; Basinga *et al.* 2011; Chandrashekar *et al.* 2014; Siapka *et al.* 2014).

Documenting potential economies of scale as well as other determinants of economic efficiency is important for several reasons. First, Kenya is the country with the largest expenditure in terms of HIV prevention activities among low- and middle-income countries (LMICs) in the world (Amico *et al.* 2012). Second, the *Getting to Zero* and *Fast Track: Ending the AIDS Epidemic* campaigns have been formulated in a time when more people will be living with HIV/AIDS, demanding dramatic increases in funding (Hecht *et al.* 2010; Institute of Medicine 2011; UNAIDS 2015b). At the same time, the new WHO guidelines for universal treatment of all persons testing positive regardless of CD4 count (UNAIDS 2015a), which have been adopted by Kenya, imply an expanded demand for resources (NASCOP 2016). In this context, major shortfalls and concerns about sustainability of international financing for health exist (Medecins Sans Frontieres 2010; Quinn and Serwadda 2011; African Union 2012; UNAIDS 2012; Kates *et al.* 2015), stressing the need for LMICs to make the best use of resources combining best practices of targeted public and private interventions (Sinanovic and Kumaranayake 2006; Hecht *et al.* 2009; Anderson *et al.* 2014). It is within this transitionary context that the prevention program in Kenya has recognized the importance of investing in an efficiency and effectiveness framework in the current KNASP (NACC 2014b) while increasing domestic financing for HIV programming (UNAIDS 2013a; AU and UNAIDS 2014). Third, there is a dearth of empirical evaluations of costs for HIV prevention with important evaluations relying on mathematical modelling (Boily *et al.* 2007; Galarraga *et al.* 2009). Lastly, the methods to measure cost and scale have developed slowly in the HIV field over the past decade with innovations still necessary to optimize program scale and economic efficiency (Kumaranayake 2008). Mathematical modelling in costing has played an important role, but the mathematical models can only predict accurately if there is empirical measurement of costs at various scales. Most of the literature has explored costs and scale in HIV prevention relying on modelling, with only few recent exceptions (Lepine *et al.* 2015); thus, the technical issues of documenting costs and their relationship with scale of HIV prevention services production remain as fertile areas of research with important policy implications.

## Methods

This study was part of the large multi-country ‘*Optimizing the Response of Prevention: HIV Efficiency in Africa*’ (ORPHEA) research project (2011–14) that was carried out in Kenya, Zambia, South Africa, Rwanda and Nigeria. The general multi-country methods are presented elsewhere (Bautista-Arredondo *et al.* 2014). The ORPHEA Kenya study ran from March 2012 to December 2013 and was developed following consultations with representatives of the National AIDS and STI Control Programme (NASCOP), the National AIDS Control Council (NACC), as well as other main HIV/AIDS stakeholders in the country. All research procedures were approved by the Kenyatta National Hospital/University of Nairobi Institutional Review Board and Northeastern University, Boston, USA.

The study sampled Government of Kenya (GOK) health service delivery points at all levels and private providers (i.e. for-profit and not-for-profit service providers, and faith-based facilities) at each level within the health system (hospitals, nursing and maternity homes, medical clinics, and dispensaries). We used multistage sampling techniques to select 56 sites for HTC, 57 sites for PMTCT and 31 sites for VMMC, for a total of 144 sites in 78 health facilities, with most facilities offering more than one intervention. Ten out of 47 counties in Kenya were purposively selected for inclusion in the study to ensure national representation. Data were collected at the district level and at each of the 144 sites. The study collected information through several avenues: interviews with facility in-charges and other relevant health staff; record verification; payslip checking; direct observation; client exit interviews; and provider vignettes. In addition, we gathered: district- and site-level characteristics, inputs to HIV service production, amount of services produced by each site, quality of services provided, service coverage, sources of funding, accountability-related characteristics and the potential demand of relevant HIV services in the same area. Cost data were collected retrospectively for the most recent year available: 2011 or 2012 (see Supplementary Technical Appendix, Section 1.6, for additional details on sampling).

Once the data were cleaned, coded and checked for inconsistencies, we calculated total annual facility costs, average costs and cost heterogeneity of producing each HIV prevention intervention as well as the determinants of management efficiency, namely key management aspects such as supervision, accountability, monitoring, incentives and governance. We used a micro-costing (ingredients) approach to estimate total variable costs as the product of the annual number of clients for each intervention times the price for each component of the HIV prevention service. Variable costs included specific items such as rapid tests, antiretrovirals for PMTCT prophylaxis, surgical circumcision kits, etc. Fixed costs included items such as utilities, capital, equipment, training and supervision, etc. We then calculated average costs by dividing the total costs incurred in the facility for each HIV prevention intervention by the total number of clients served for each particular service in that facility (Drummond 2005). A combination of space and time allocation was used to apportion costs to tasks jointly producing more than one HIV prevention service (Roberts 2006). (See Supplementary Technical Appendix, Section 1.7.1, for additional details on measuring costs).

The costs were collected in current Kenyan shillings, and transformed into US dollars at the constant exchange rate of 88.9 shillings per USD for the year 2011 (Central Bank of Kenya 2015). The dependent variable (average costs) was log transformed to more closely approximate a normal distribution, be able to apply linear regression methods, and to be able to interpret the scale coefficients as an elasticity (Manning and Mullahy 2001; Zhou *et al.* 2001). For the statistical analysis, we used linear regression methods. Based on the theory and previous literature (Over 1986; Dandona *et al.* 2005; Guinness *et al.* 2005; Boily *et al.* 2007; Marseille *et al.* 2007, 2012; Chandrashekar *et al.* 2010) we included a large selection of potential predictors of unit costs.

In addition, we included the following measures relevant to evaluating costs and efficiency determinants (Kasymova *et al.* 2009; Basinga *et al.* 2011; Preyra and Pink 2001; Chandrashekar *et al.* 2014; Siapka *et al.* 2014): incentives for staff performance as well as at the facility level; questions on the ownership of the hospitals and/or facilities variables related to service-integration and task-shifting; and variables related to supervision and outreach costs. (Details on the selected variables explored are presented in Supplementary Technical Appendix Exhibit A2). In addition, two indexes of quality were estimated with principal components analysis: Competence and Performance.

For the log-transformed linear regression models, we first analysed the number of clients per year (scale) as the main predictor; and then we analysed the full model adjusting for other types of variables such as those related to the service delivery model as well as the management of HIV prevention services at the facility level. We adopted an accounting identity approach to characterize the relationship between cost and its determinants (Meyer-Rath and Over 2012), assuming that scale effects were generated at the facility level only and that facility and contextual characteristics increased or decreased cost multiplicatively. Taking logarithms allowed us to estimate the identity equation via ordinary least squares regression and test for the sign of the regression coefficients, such as scale effects. (Supplementary Technical Appendix, Section 3, presents the model derivation and equations).

The aim of the model was to quantify the correlation between unit costs and scale of production, where scale was defined as the number of clients served with the specific HIV prevention service produced. The main determinants included were based on general microeconomic theory (Perloff 2017) and previous literature (Bautista-Arredondo *et al.* 2014; Marseille *et al.* 2004) adapted to HIV prevention services production so that we specifically included the most relevant factors as follows. The annual number of clients served measured the scale of production: This was done at each step of the service cascade, such that for testing for example, we first measured all clients tested, and then we measured the number of clients who tested HIV positive. We also included number of supervisions received because oversight may be important in determining efficiency. Similarly, we included measures of community-based or outreach operations, which may be more costly; or whether the facility targeted the testing of populations most at risk (PMAR), which again may require additional economic resources. We also included whether the facility had a community advisory council because appropriate guidance and leadership may affect efficiency; as would do incentives: whether staff could receive rewards for good performance. Finally, we included a measure of whether the facility performed task shifting, meaning that HIV prevention services may be produced by delegating specific tasks to less specialized personnel. The specific variables in the final model were also chosen for their initial statistical significance (*P *<* *0.10) as well as their overall contribution for the model’s explanatory power as measured by the overall model significance (*F* test) and the *R*-squared. We used the term predictor instead of independent variable to emphasize that we did not have an experimental design, so the model measured only associations given by the direction and magnitude of the coefficients from linear regression. The main component of unit costs were staff salaries which we measured using allocations based on full time equivalents (FTE) devoted to specific HIV prevention services (see Supplementary Technical Appendix, Section 2.1). Thus, this method implicitly accounted for capacity because some facilities may have more employees, which served more or less clients depending on various aspects of (technical) efficiency, while holding other characteristics (such as quality) constant.

## Results

Table 1 presents the dependent variables: the average costs per client. For HTC, the average cost per client tested was $7, while the average cost per client tested and found HIV-positive was $146. For PMTCT, the average cost per client tested was $59, while the average cost per client tested and found HIV-positive was $674. For VMMC, the average cost per procedure completed was $66.

**Table 1.** Dependent variables: average cost per client across selected indicators of the HIV prevention service cascade in Kenyan facilities, 2011–2012

	*N*	Mean	95% CI		Weighted mean	Median	SD	IQR
HTC								
Cost per client tested	56	7.4	5.5	9.2	6.5	4.8	7.1	5.9
Cost per client tested and positive	56	145.9	62.6	229.2	80.2	54.9	318.0	74.7
PMTCT								
Cost per client tested	57	58.7	36.8	80.6	48.5	34.6	84.3	59.0
Cost per client tested and positive	51	673.5	388.8	958.1	775.8	256.3	1,037.1	594.5
Cost per client on ART	31	1,385.0	64.7	2,705.2	1,261.6	274.8	3,750.5	701.2
VMMC								
Cost per procedure	33	66.3	39.5	93.1	41.1	42.4	78.6	51.1

Weighted mean according to total annual patient volume.

CI, confidence interval; SD, standard deviation; IQR, interquartile range; ART, Antiretroviral therapy; HTC, HIV testing and counselling; PMTCT, Prevention of mother-to-child transmission; VMMC, Voluntary medical male circumcision.

Table 2 shows the cost predictors as characteristics of the service delivery model and management indicators affecting the costs of HIV prevention interventions in Kenya by type of facility (hospital vs non-hospital). The survey included a total of *N* = 56 facilities that provided HTC services to an average of 4235 clients per year in each facility (last columns). There were differences by type of facility: hospitals had a greater number of HTC clients than non-hospital facilities (5244 vs 3071); and hospital’s HTC staff were also more likely to receive rewards for good performance in comparison to staff in non-hospital facilities (40 vs 19%). Among the 57 facilities selected for PMTCT services, an average of 864 clients were tested annually in each facility; and hospitals were also more likely to have PMTCT staff who can receive rewards for good performance in comparison to non-hospital facilities (47 vs 20%). In the 33 facilities providing VMMC, an average of 869 VMMC procedures per year were conducted in each facility. Hospitals were also more likely to have VMMC staff who can receive rewards for good performance in comparison to non-hospital facilities (50 vs 17%).

**Table 2.** Predictors: service delivery and management indicators for HIV prevention interventions by facility type, Kenya, 2011–12

	Hospital				Non-hospital				*P*-value	Total			
	*N*	*M*	95% CI		*N*	*M*	95% CI			*N*	*M*	95% CI	
HTC													
Annual number of clients tested	30	5,244	3,553	6,934	26	3,071	1,565	4,576	0.069	56	4,235	3,065	5,404
Annual number of clients tested and positive	30	410	276	544	26	338	164	513	0.522	56	377	269	484
Number of supervisions received in 2011	30	13	9	17	26	18	4	32	0.447	56	16	9	22
Facility performs community based testing	30	0.40	0.22	0.58	26	0.27	0.10	0.44	0.311	56	0.34	0.21	0.46
Facility targets testing (PMAR—symptoms)	30	0.23	0.08	0.39	26	0.15	0.01	0.30	0.464	56	0.20	0.09	0.30
Facility has a community advisory council	30	0.30	0.13	0.47	26	0.38	0.19	0.58	0.514	56	0.34	0.21	0.46
Staff can receive rewards for good performance	30	0.40	0.22	0.58	26	0.19	0.04	0.35	0.095	56	0.30	0.18	0.43
Facility performs task shifting	30	0.57	0.39	0.75	26	0.58	0.38	0.77	0.940	56	0.57	0.44	0.70
Total number of FTE (clinical)	30	4.61	2.94	6.27	26	3.04	1.77	4.32	0.159	56	3.88	2.80	4.96
Total number of FTE (non-clinical)	30	0.69	0.18	1.20	26	0.69	0.29	1.08	0.987	56	0.69	0.36	1.02
PMTCT													
Annual number of clients tested	32	1,033	662	1,404	25	648	259	1,037	0.169	57	864	593	1,136
Annual number of clients tested and positive	29	63	34	92	22	67	37	96	0.859	51	64	44	85
Number of supervisions received in 2011	32	14	10	18	25	12	6	17	0.479	57	13	10	16
Facility targets testing (PMAR—symptoms)	32	0.03	−0.03	0.09	25	0.00	0.00	0.00	0.382	57	0.02	−0.02	0.05
Funding linked to facility performance	32	0.31	0.15	0.48	25	0.20	0.04	0.36	0.347	57	0.26	0.15	0.38
Staff can receive rewards for good performance	32	0.47	0.29	0.64	25	0.20	0.04	0.36	0.035	57	0.35	0.23	0.48
Facility performs task shifting	32	0.47	0.29	0.64	25	0.56	0.36	0.76	0.503	57	0.51	0.38	0.64
Total number of FTE (clinical)	32	7.70	5.32	10.07	25	3.78	2.55	5.00	0.011	57	5.98	4.46	7.49
Total number of FTE (non-clinical)	32	1.41	0.63	2.19	25	0.75	0.28	1.22	0.188	57	1.12	0.63	1.61
VMMC													
Annual number of clients tested	10	855	611	1,098	23	875	495	1,255	0.946	33	869	597	1,141
Number of supervisions received in 2011	10	15	8	22	23	17	2	32	0.869	33	17	6	27
Facility has a community advisory council	10	0.30	0.00	0.60	23	0.26	0.08	0.44	0.824	33	0.27	0.12	0.43
Facility offers male reproductive health services	10	0.90	0.70	1.10	21	0.76	0.58	0.95	0.380	31	0.81	0.67	0.95
Staff can receive rewards for good performance	10	0.50	0.17	0.83	23	0.17	0.02	0.33	0.056	33	0.27	0.12	0.43
Facility performs task shifting	10	0.30	0.00	0.60	23	0.30	0.11	0.50	0.981	33	0.30	0.14	0.46
Total number of FTE (clinical)	10	4.02	2.52	5.52	23	2.71	2.07	3.35	0.072	33	3.11	2.45	3.77
Total number of FTE (non-clinical)	10	1.43	0.41	2.46	23	0.80	0.35	1.25	0.204	33	0.99	0.55	1.44

This table presents proportions unless otherwise stated. The non-hospital category includes health centres, dispensaries and clinics. HTC, HIV testing and counselling; PMTCT, Prevention-of-mother-to-child-transmission; VMMC, voluntary medical male circumcision; *N*, number of sites; CI, confidence interval. The *P*-values are probability values for statistical comparison tests between hospital and non-hospital facilities. Annual number of supervisions received in 2011 was computed as the sum of self-reported supervisory visits by donors and national, provincial or district governments during the costing year. Facility performs community based testing is a binary indicator of facilities that reported offering testing and pre/post-test counselling for individuals, couples or groups. PMAR, Populations-most-at-risk (indicates if they reported offering HIV testing based on client screening for symptoms or having profiles characteristic of high-risk populations. Funding linked to performance indicates facilities that reported that at least part of the funding is tied to one or more of the following criteria: inputs management, inventory management, quality of care or patient volume. Staff can receive rewards for good performance indicates if the facilities reported established mechanisms such as bonuses, certificates, verbal recognition, training, preferential rotation or time-off. Facility performs task shifting indicates if care activities have delegated to nurses and other health staff, based on the time-allocation component of the study. Facility offers male reproductive health services indicates if they reported performing detection and treatment of health issues such as fertility or erectile dysfunction. FTE denotes full-time equivalent. Clinical staff includes doctors, nurses and other health staff (e.g. counsellors); non-clinical staff includes clerical, managerial and other support staff (e.g. guards).

**Figure 1.**
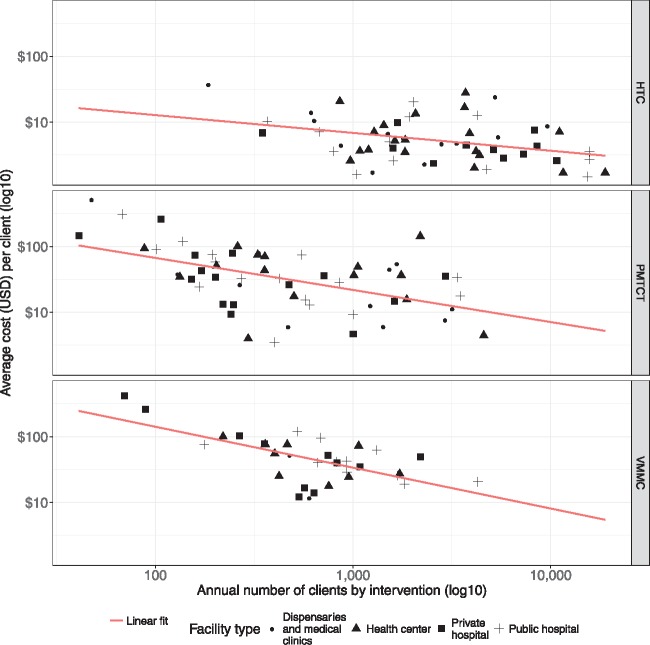
Average costs per client and number of clients, by intervention type

We now present graphical results. Figure 1 plots the relation between the average costs per client and the total number of clients by intervention type (HTC, PMTCT and VMMC), differentiating also by type of facility (public vs private hospital, as well as health centre, dispensaries and clinics). As the scale of production increased, the per-client costs declined for all interventions. Analysing the detailed results (Supplementary Tables S1–S3), we see that doubling the number of clients tested reduced the cost of per PMTCT client by 50%; while doubling the number of clients reduced the cost of per VMMC procedure by 45%. The scale coefficient for HTC was also negative (−0.18) but was not statistically significant. (We repeated the process using fully adjusted multivariate models which have higher predictive value, as given by higher *R*-square coefficients. Supplementary Figure A1 shows that the size of the scale effect is consistent between the bivariate and multivariate models).

**Figure 2.**
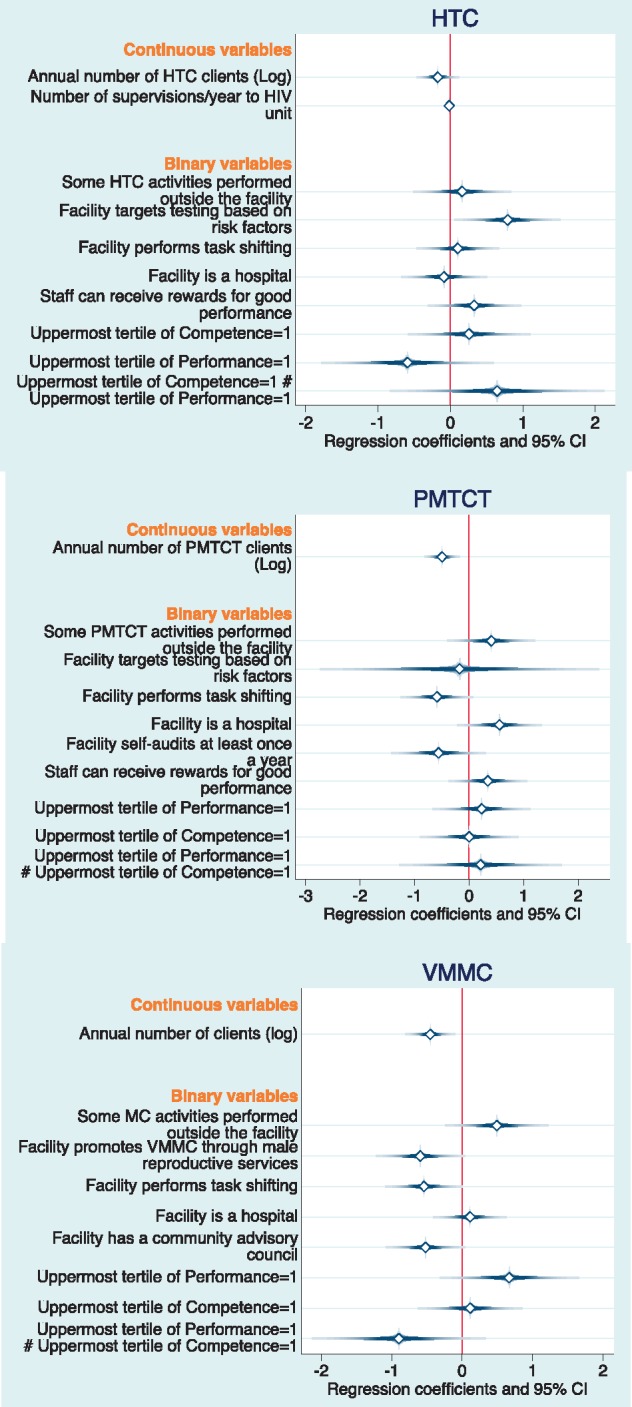
Multiple regression coefficients for Log of cost per HTC, PMTCT and VMMC client

Figure 2 summarizes the coefficients for the other predictors of the average costs (detailed results are presented in Supplementary Tables S1–S3). For HTC, the factor most strongly correlated with costs was whether the facility targets testing based on risk factors (*β *= 0.79; CI 95% 0.24–1.34). The HTC costs model including scale explained about a fifth of the variation in the average costs (*R*-squared = 0.22). For PMTCT, costs decreased if the facility performed task shifting (*β* = −0.59; CI 95% −1.09 to −0.09) and also if the facility performed audits at least once per year (*β* =−0.56; 95% CI −1.21 to 0.09). On the other hand, PMTCT costs increased if the facility was a hospital (*β *= 0.56; 95% CI −0.03 to 1.14). The final PMTCT model including scale explained over a third of the variation in the average costs (*R*-squared = 0.35). For VMMC, costs decreased if the facility promoted the procedure through the male reproductive services (*β* = −0.59 95% CI −1.06 to −0.13); if the facility performed task shifting (*β* = −0.54; 95% CI −0.94 to −0.14); and if the facility had a community advisory council (*β* = −0.52; 95% CI −0.93 to −0.11). On the other hand, VMMC costs increased if some of the activities were performed outside the facility (*β *= 0.49; 95% CI −0.04 to 1.03), and for the facilities that had the highest performance (*β *= 0.67; 95% CI −0.06 to 1.40). Further, there was an interaction effect whereby facilities that were both the best in terms of competence and performance for VMMC had substantially lower costs (*β* = −0.90; 95% CI −1.81 to 0.01). The final VMMC costs model including scale explained most of the variation in the average costs (*R*-squared = 0.59).

## Discussion

This is, to our knowledge, the first article examining predictors of HIV prevention costs at the national level in sub-Saharan Africa. Several points merit discussion. First, most studies in the past have been based on smaller samples, localized interventions and non-standard data collection methods (Galárraga *et al.* 2009; Menzies *et al.* 2012; Bautista-Arredondo *et al.* 2014). Second, this article reports the associations between the average cost of each prevention intervention (HTC, PMTCT and VMMC) and scale, as one of the most widely discussed determinant in the literature (Johns and Baltussen 2004; Kumaranayake 2008), as well as other determinants of average costs. In addition, this article also explores combination of HIV prevention services as well as integration with other services. For all interventions, we found point estimates consistent with evidence of economies of scale: Average cost tends to decrease as facilities serve more clients. Doubling the number of clients was correlated with 18% decrease in HTC costs, 51% for PMTCT and 45% for VMMC (though the HTC estimate was not significant). Third, task shifting (i.e. using qualified lower level staff instead of physicians) correlates with lower unit costs for PMTCT and VMMC, but not for HTC. This result seems logical as the tasks for PMTCT and VMMC may be more amenable to be shifted to personnel with fewer formal qualifications than HTC, which is already conducted by staff with minimum levels of formal training. The literature has provided evidence of the potential use of task-shifting alcohol interventions for HIV-positive persons in Kenya (Galárraga *et al.* 2017), and for PMTCT and VMMC in other countries as well (Fieno 2008; Lehmann *et al.* 2009; McCollum *et al.* 2010; Fulton *et al.* 2011; Aliyu *et al.* 2013; Siapka *et al.* 2014). In addition, for VMMC there was a positive association between unit costs and performing activities outside the facility (e.g. mobile units). This result may be explained by the increased costs associated with outreach (Larson *et al.* 2015).

Fifth, the costs of PMTCT, HTC and VMMC are largely consistent with the previous literature. The cost per HTC client of $7.4 in Kenya suggests that costs have decreased over time as scale has increased when compared to a previous estimation of $16 per client a decade earlier (Forsythe *et al.* 2002). The median PMTCT cost per client on ART of $275 is higher than what was found for Zambia for another project ($185) (Scott *et al.* 2013), and will complement estimates that have relied on modelling (Sweat *et al.* 2004; Gopalappa *et al.* 2014). The costs observed in Zambia may have been lower possibly because Scott *et al.* (2013) relied on a convenience sample while this article presents nationally representative results for Kenya. The cost of $66 per VMMC procedure is comparable to previous estimates of $59–74 in Swaziland, and $56–61 in Zimbabwe (Edgil *et al.* 2011; Njeuhmeli *et al.* 2014). Similarly, in Uganda the VMMC costs were $34 at fixed sites and $61–72 in mobile sites (Larson *et al.* 2015).

The results also show that unit costs tend to be higher in hospitals for PMTCT, but not for the other interventions (Supplementary Tables S1–S3). Two offsetting forces may be at work in this observed relationship relating to hospitals. The first may be that hospitals have overall lower unit costs because of economies of scale. As was seen in Table 2, in comparison to non-hospital based facilities, hospitals had larger numbers of clients per year for HTC (though not for PMTCT and VMMC). At the same time, a second set of variables may make hospitals less efficient as they were more likely to have funding linked to facility performance for PMTCT. Notably, incentives for staff with good performance were not associated with changes in costs. This result is unexpected as incentives for good performance usually add to the unit costs (Saronga *et al.* 2014).

For VMMC other factors associated with lower average costs were the presence of male reproductive health services at the facility, as well as the existence of a community advisory board. The former result provides some evidence of economies of scope (and/or integration) as related to VMMC facilities, while the latter result may be related to overall facility supervision and oversight (Gray *et al.* 2013; Siapka *et al.* 2014).

Our results suggest that there is a potential to increase efficiency within the current constraints of the health system in Kenya, both financial and structural. Specifically, we found that scale is important even across facility types, and not just comparing hospitals with clinics. In light of these results, it is important to think about scale not only as a given factor, which in many circumstances it is, but also to give importance to demand creation activities at the facility level. Our results suggest that this type of investment will probably be very productive. It may also be important to consider economies of scale when determining the size or capacity of health facilities and when selecting their location and size. The results also suggest the importance of evaluating excess supply, such as the extent to which current levels of structure and staffing are not being used to their optimum potential. This may become increasingly important to consider at the policy level as devolution of health deepens (Kibui *et al.* 2015). Already, counties are allocating varied financing for health programming (Maina *et al.* 2016), and some guidance may be needed towards achieving efficiencies.

In terms of strengths, this article contributes a specific example of applying a micro-costing approach to HIV prevention services and modelling a unit cost function in terms of its main predictors. The main weakness may be that we can only observe associations given the cross-sectional nature of the data. Thus, more rigorous research is needed to attempt to measure causal relationships in the future. In addition, there are other limitations. First, our modelling choice of a cost accounting identity imposes arithmetical consistency, which is useful for short-run budgeting discussions and enables us to make projections of incremental policies such as scale-up in coverage. However, our models are agnostic with respect to technology, as opposed to flexible cost functions. Thus, other policy concerns such as substitution between health inputs or the impact of economies of scope would necessitate other econometric approaches, which would require more degrees of freedom than those available in our data. A second concern is that the study did not account for all of the potential variables that can affect unit cost variations; other constraints, different from technology and competence, may also explain inefficiency. Finding the right balance in key management aspects such as supervision, accountability, monitoring, incentives and governance remains a challenge, especially in the fully devolved county health functions. Additional exploration of facility-level and county-level management practices and standards that explain variability in efficiency is needed. Third, our study provides evidence—and identifies gaps—on the efficiency and costs of HIV prevention services for a specific cross-section at a particular time point. Ideally, this type of information should be provided on a continued basis and even in real-time to decision makers and managers at all levels in the health system; thus, more regular evaluations are recommended. Although the results may be applicable to other settings and times with similar set of circumstances these findings are specific to Kenya. Finally, the *R*-squared for the intervention models, given that we have cross-sectional data, were only modestly high and therefore conclusions need to be made with caution.

## Conclusion

The analysis has established that volume of service or scale of output explains considerable variation in unit costs for HIV prevention activities, but not all. This implies that an increase in the number of clients in all facilities and particularly in the lower level facilities can lead to declines in costs. Expanding the volume of services can improve levels of efficiency in the HIV prevention response particularly for PMTCT and VMMC. Other factors associated with decreased costs were: task-shifting, community oversight and service integration. Factors associated with increased costs were: hospital-based services; and outreach efforts. Aside from increasing production scale, HIV prevention costs may be further contained by using task shifting for PMTCT and VMMC. In contrast, targeted testing for HTC may require more resources. ORPHEA provides the first national-level evidence base for HIV prevention costs and their determinants for an African country, Kenya.

## Supplementary Material

Supplementary Material
