# Supplementary material for: HIV prevention costs and their predictors: evidence from the ORPHEA Project in Kenya
Source: Health Policy Plan. 2017 Sep 26;32(10):1407–16. doi: 10.1093/heapol/czx121 (PMC5886164; doi:10.1093/heapol/czx121)
Supplement: Supplementary Material [file supplementary_material_czx121.docx]

**SUPPLEMENTARY MATERIAL**

**for**

**HIV prevention costs and their predictors:**

**Evidence from the ORPHEA project in Kenya**

**TABLE OF CONTENTS**

Supplementary Tables S1-S3………………………………………………pgs. 2-3

Supplementary Figure A1………………………………………………….pg.4

Supplementary Technical Appendix…………………………………….…pgs.5-17

| **Supplementary Table S1** HIV Testing and Counseling Average Cost Model, Kenya, 2011-12 | | | | |
| --- | --- | --- | --- | --- |
|  | (1) | (2) |  |  |
| VARIABLES | Coeff. | 95% CI |  |  |
| Annual number of HTC clients (Log) | -0.18 | -0.39 - 0.04 |  |  |
| Number of supervisions/year to HIV unit | -0.02** | -0.03 - 0.00 |  |  |
| Some HTC activities performed outside the facility | 0.16 | -0.35 - 0.67 |  |  |
| Facility targets testing based on risk factors | 0.79*** | 0.24 - 1.34 |  |  |
| Facility performs task shifting | 0.10 | -0.33 - 0.53 |  |  |
| Facility is a hospital | -0.09 | -0.54 - 0.36 |  |  |
| Staff can receive rewards for good performance | 0.33 | -0.15 - 0.81 |  |  |
| Uppermost tertile of Competence = 1 | 0.26 | -0.38 - 0.89 |  |  |
| Uppermost tertile of Performance = 1 | -0.60 | -1.49 - 0.30 |  |  |
| Uppermost tertile of Performance = 1 x Uppermost tertile of Competence = 1 | 0.64 | -0.47 - 1.76 |  |  |
| Constant | 2.90*** | 1.23 - 4.58 |  |  |
| Observations | 56 |  |  |  |
| Adjusted R-squared | 0.22 |  |  |  |

**Notes**: **p*<0.10 ***p*<0.05 ****p*<0.01; Coeff is the coefficient from the log-linear regression in column (1) and the 95% confidence interval (CI) in column (2). HTC is HIV Testing and Counseling.

| **Supplementary Table S2** Prevention of Mother-to-Child Transmission Average Cost Model Kenya, 2011-12 | | | | | | | | |  |
| --- | --- | --- | --- | --- | --- | --- | --- | --- | --- |
|  |  |  | |  | |  |  |  | |
|  | | | (1) | | (2) | | |  |  |
| VARIABLES | | | Coeff. | | 95% CI | | |  |  |
| Annual number of PMTCT clients (Log) | | | -0.50*** | | -0.74 - -0.25 | | |  |  |
| Some PMTCT activities performed outside the facility | | | 0.40 | | -0.20 - 1.01 | | |  |  |
| Facility targets testing based on risk factors | | | -0.18 | | -2.10 - 1.74 | | |  |  |
| Facility performs task shifting | | | -0.59** | | -1.09 - -0.09 | | |  |  |
| Facility is a hospital | | | 0.56* | | -0.03 - 1.14 | | |  |  |
| Facility self-audits at least once a year | | | -0.56* | | -1.21 - 0.09 | | |  |  |
| Staff can receive rewards for good performance | | | 0.34 | | -0.20 - 0.89 | | |  |  |
| Uppermost tertile of Performance = 1 | | | 0.23 | | -0.44 - 0.90 | | |  |  |
| Uppermost tertile of Competence = 1 | | | 0.00 | | -0.68 - 0.68 | | |  |  |
| Uppermost tertile of Performance = 1 x Uppermost tertile of Competence = 1 | | | 0.21 | | -0.91 - 1.33 | | |  |  |
| Constant | | | 6.61*** | | 5.01 - 8.21 | | |  |  |
| Observations | | | 57 | |  | | |  |  |
| Adjusted R-squared | | | 0.35 | |  | | |  |  |

**Notes**: **p*<0.10 ***p*<0.05 ****p*<0.01; Coeff is the coefficient from the log-linear regression in column (1) and the 95% confidence interval (CI) in column (2). PMTCT is prevention of mother-to-child transmission

| **Supplementary Table S3** Voluntary Medical Male Circumcision Average Cost Model Kenya, 2011-12 | | | | |
| --- | --- | --- | --- | --- |
|  | (1) | (2) |  |  |
| VARIABLES | Coeff. | 95% CI |  |  |
| Annual number of clients (log) | -0.45*** | -0.71 - -0.19 |  |  |
| Some VMMC activities performed outside the facility | 0.49* | -0.04 - 1.03 |  |  |
| Facility promotes VMMC through male reproductive services | -0.59** | -1.06 - -0.13 |  |  |
| Facility performs task shifting | -0.54** | -0.94 - -0.14 |  |  |
| Facility is a hospital | 0.11 | -0.27 - 0.49 |  |  |
| Facility has a community advisory council | -0.52** | -0.93 - -0.11 |  |  |
| Uppermost tertile of Performance = 1 | 0.67* | -0.06 - 1.40 |  |  |
| Uppermost tertile of Competence = 1 | 0.11 | -0.43 - 0.66 |  |  |
| Uppermost tertile of Performance = 1 x Uppermost tertile of Competence = 1 | -0.90* | -1.81 - 0.01 |  |  |
| Constant | 7.06*** | 5.17 - 8.95 |  |  |
| Observations | 31 |  |  |  |
| Adjusted R-squared | 0.59 |  |  |  |

**Notes**: **p*<0.10 ***p*<0.05 ****p*<0.01; Coeff is the coefficient from the log-linear regression in column (1) and the 95% confidence interval (CI) in column (2). VMMC is Voluntary Medical Male Circumcision.

**Supplementary** **Figure A1** Average costs per client: Bivariate vs. multivariate models

**Note**: We implemented the concept of “regression anatomy” using the *reganat2* Stata command. The figure shows that the size of the scale effect is consistent between the bivariate and multivariate models.

**SUPPLEMENTARY TECHNICAL APPENDIX**

**1.1 Defining efficiency**

Following microeconomic theory, the building block of the discussion on efficiency is the production function, which describes a technical relationship between inputs and outputs for a given technology. Panel I of Exhibit A1 describes this concept graphically for a hypothetical HTC program. A production function thus, describes for any level of inputs (*X_1_*) the resulting amounts of outputs produced (*Y_1_*). Assuming that no resources are wasted, the production function describes production at the most efficient levels possible, given the current technology.

**Supplementary Exhibit A1** Two hypothetical production functions for HTC

*X_1_*

*Y_1_*

*X_1_*

*Y_1_*

*Y_2_*

A

B

C

D

***II***

*Input X*

*Input X*

*HTC*

*HTC*

***I***

E

**1.2 Defining technical efficiency**

From the cost side, we define technical efficiency as the minimum unit cost per service delivered at given levels of outputs and given input prices and other exogenous characteristics of the “market” or context in which the facility operates. This concept is illustrated in panel II of Exhibit A1. The production function depicted is the same theoretical function as the one showed in panel I, but in this case each dot in the Figure represents a specific facility for which empirical data exist. Facilities A through D are facilities operating with efficiency at different levels of output (or scale of production). Facility E on the other hand, is performing inefficiently because it is using more inputs (*X_1_*) than it should, given its level of output (*Y_1_*). The horizontal arrow represents the amount of resources wasted.

In the markets of goods and services produced by the private sector, competition among producers, combined with their objective of maximizing profits, minimize the existence of inefficiencies. Any firm (such as facility E in Exhibit A1), producing at significantly higher costs than its competitors, will eventually lose any chance of remaining in the market.

However, private sector firms could fail to supply public goods and services at a satisfactory quality level. For example, in the context of HIV prevention, “firms” may vary in their goals, and thus not all firms may perform in a manner to maximize their profits. Other priorities, which reflect what they want to achieve may be important, such as maximizing utility or quality (e.g., good quality results for a small pool of clients, high visibility in their communities). In many cases, the income of these organizations may not depend on maximizing profits or on any measure of performance. Particularly important for HIV prevention, severe demand-side constraints exist. For example, in order to provide adequate PMTCT services, a minimum level of investment is necessary, and this level of investment might be extremely high for certain geographical areas where the demand for this type of service will be very small.

**1.3 Measuring efficiency**

To estimate efficiency, using both cost and production perspectives, we collected comprehensive data at the facility level on three dimensions of the production of HIV prevention services. (1) Outputs, defined as all services produced at a given time. (2) Inputs, defined as everything that the facility employs to produce these services, including purchased and donated inputs at market prices. (3) Facility and contextual characteristics also referred to as “organizational” characteristics, describing the process and context in which production decisions are made. Using this information, we estimated cost and production functions to obtain a measure of efficiency (or inefficiency) for each facility.

There are different methodological alternatives to estimate (in)efficiency; however, the intuition is the same. First, the average unit cost per output and the average productivity is estimated for each facility (represented by each dot in Exhibit A1, panel II). Second, an empirical frontier of productivity is estimated. This empirical estimate of the frontier describes the observed relation between inputs and outputs in the sample (i.e. the technology observed), but it also represents the maximum productivity (or the minimum cost) achievable at any given level of output (represented by the gray line in Exhibit A1, panel II). Third, the distance between the observed level of productivity (or input cost) and the production frontier is measured. This distance, whether it is a production distance or an input cost distance, is the empirical estimate of inefficiency (in Exhibit A1, panel II, these are represented by the vertical and horizontal arrows, respectively, for facility E).

**1.4 Explaining (in)efficiency**

One useful approach to explain heterogeneity in efficiency levels among a sample of firms focuses on explaining the inefficiency levels observed in the sample (i.e., the distance between any given observation and the production frontier). Using regression techniques, the objective is to explain the variability in the distance with relevant facility-level characteristics. These characteristics describe decisions made at the facility level (the organizational and managerial structure of the facilities are very relevant for this process), as well as constraints faced by the facility (the context, demand for services, and donor restrictions and requirements are the important facets to consider in this case). The following three categories of variables describe these characteristics in more detail, although they are not necessarily mutually exclusive.

• Outputs. Examples of output related variables that can explain inefficiency in a given facility include: quality, which is typically resource-consuming; complexity, which is in many cases imposed from the demand side, for instance by the type and localization of clients; and scope of production, that is whether the organization produces more than one type of service.

• Inputs. Here, we are interested in which inputs are used and how. Usually, this category of variables has to do with decisions made at the facility, for example: how inputs are shared in different processes of production, which specific inputs are purchased and used for production, and how labor inputs are managed and which incentives for performance they face.

• Facility and contextual characteristics. In this category, we include two types: contextual determinants and policy- and management-level determinants. First, contextual determinants related to outputs and inputs include funding source(s) of the organization and other contextual determinants, such as location (urban or rural), competition, facility type (hospital or clinic), HIV prevalence, ownership (public or private), supply of services (to what extent electricity and water work), and size of demand. Second, policy- and management-level determinants include those related to outputs or inputs (e.g. monitoring and evaluation intensity, procurement autonomy, manager’s training, user fees) and other policy levers, such as management (e.g. supervision, incentives), information system quality and use, and accountability to the local community, patients, board, government, or donors.

## Study population

The ORPHEA Kenya study population consists of nationally representative Government of Kenya (GOK) and non-government (NGOK) health facilities or sites that offered the following HIV prevention services: HTC, PMTCT, and VMMC. The study sampled GOK health service delivery points at all levels^^[[1]](#footnote-1)^^ and private providers (i.e., for-profit and not-for-profit service providers, and faith-based facilities) at respective levels within the health system (hospitals, nursing and maternity homes, medical clinics, and dispensaries).

## Sample size determination and sampling strategy

To ensure that the study was powered to detect significant association between potential explanatory variables and the performance of a unit, we estimated the sample size ($n$) to be sufficient to identify when the Pearson correlation coefficient is different from zero.^[[2]](#footnote-2)^ We based our estimations on the correlations between costs and scale derived in the PANCEA study.^[[3]](#footnote-3)^ Based on the sample size suggested by different correlation coefficients, our target sample size was set to 40 sites for each intervention (HTC, PMTCT and VMMC), and as consequence, the target sample size for all of the study was set to 120 sites. We expected to identify correlation coefficients greater than 0.45 for associations between sites’ characteristics and their performance for each intervention, and correlation coefficients greater than 0.25 for associations between sites’ characteristics and their performance across all the interventions.

Multistage sampling techniques were used to select counties and health facilities for the Kenya sample. Local HIV prevalence, facilities providing the same service(s) nearby, as well as security considerations were also taken into account when constructing the study sample. With respect to local HIV prevalence, we adopted a strategy of proportional sampling whereby higher weighting was given to localities with higher prevalence.

### Stage 1: Selection of counties^^[[4]](#footnote-4)^^

Ten out of 47 counties in Kenya (representing 32% of the total population) were purposively selected for inclusion in the study to ensure national representation. These counties included: Nairobi, Homabay, Kisumu, Kakamega, Uasin Gishu, Nakuru, Nyeri, Embu, Kitui and Mombasa. County selection was based on the following criteria: i) security considerations, which prompted exclusion of the North-Eastern region;^^[[5]](#footnote-5)^^ ii) existence of a level 5 health facility; iii) joining of counties when the pool of health facilities was not large enough to sample from it and iv) selection of two counties to represent the South and North of the expansive Rift Valley region.

### Stage 2: Selection of health facilities

The number of health facilities to be sampled from each selected county was based on a weighting system that ensured that counties with higher HIV prevalence were overrepresented. For VMMC, the sites were located in Kisumu and Homabay Counties, where the intervention was primarily being implemented. For HTC and PMTCT the sample was drawn from the 10 counties in sample.

Once the number of health facilities per county and intervention was decided, all operating facilities from the selected counties were listed and stratified within each county by: ownership (public or private) and level of service provision (levels 2 to 4). Within each stratum, facilities were randomly selected. Notably, selection of facilities from levels 2, 3 and 4 ensured representation of both urban and rural sites, as level 4 facilities are predominantly urban while levels 2 and 3 are predominantly rural. Facilities with integrated services as opposed to stand-alone services were prioritized during selection. In addition, in each county a level 5 facility was included as these offered all the HIV prevention services of interest, and were also referral facilities in the regions with comprehensive care centers.

During data collection we encountered cases of i) miss-reporting (i.e., different interventions offered at a site compared to the information reported in the sampling frame), ii) non-response (i.e., facility declined participation or was not operational), and iii) incomplete data (cost or output data were not available at the facility for six months or more). In these cases, we employed the following replacement strategy. First, we searched the sampling frame starting at row 1 until a similar facility in the same district was found. If no other facility was available in the same district, the procedure was repeated, dropping the restriction that the facility should belong to the same district and county.

## Data collection instruments

We developed standardized facility survey instruments based originally on instruments from the PANCEA project. The development and finalization of these instruments involved the Mexican National Institute of Public Health (INSP) team, the Kenya country team as well as relevant partners, including the World Bank, Elizabeth Glaser Pediatric AIDS Foundation (EGPAF), and Population Services International (PSI). We developed a set of 13 questionnaires, which were administered to a facility in-charge, or management staff and clients. Instruments included data abstraction forms for capturing relevant quantitative information from project records and reports, particularly for HIV service utilization indicators. Other data sources included: reporting forms, electronic databases, electronic or written medical records, logbooks, registers, receipts, stock cards, facility accounting records, payroll records, and performance reports. For some data items (e.g., service quality), structured interview forms were used to guide spontaneous responses obtained from interviews with providers and clients of HIV services. All instruments were administered in a quiet private place after obtaining informed consent from each study participant. The study instruments were designed to collect the following types of data:

1. District-level characteristics such as district-level HIV prevalence, number of facilities in the district, district-level training and supervision, and supplies distributed to facilities from the district level.
2. Facility-level characteristics such as range of services provided (e.g., different HIV services, inpatient services), number of beds, number of years the facility has been in operation, location, service hours, and more.
3. Inputs for service production including physical assets (e.g., facility size and age), labor (e.g., staff hours, wages, and benefits by skill category), supplies, maintenance, and utilities.
4. Outputs such as the amount of services produced (e.g., the numbers of clients for each service, counselling sessions completed, and condoms distributed).
5. Service quality in terms of both structure (i.e., the ability of a provider to deliver a certain level of service quality, particularly with certain supplies and equipment) and process (i.e., providers’ practice and effort).
6. Management characteristics including the legal and ownership structure of the facility, types of accountability mechanisms for staff and management, incentives and sanctions used by the management, and the institutional environment in which the facility operates.

The Supplementary Exhibit A2 presents some selected and illustrative questionnaire items.

**Supplementary Exhibit A2** Questionnaire (selected items)

| Variable name | Questionnaire item | Applicable interventions |
| --- | --- | --- |
| Facility targets testing (PMAR - symptoms) | Facility offers HIV tests based on client screening for symptoms or having profiles characteristic of high-risk populations | HTC, PMTCT |
| Facility performs community based testing | Facility offers testing, and pre/post-test counseling for individuals, couples and groups | HTC |
| Facility performs task shifting | Facility employs medical officers or similar (instead of physicians) to provide the intervention | HTC, PMTCT, VMMC |
| Number of supervisions received in the last year | Number of supervisions received from government and donors during the costing year | HTC, PMTCT, VMMC |
| Facility has a community advisory council | A community advisory council exists for this facility | HTC, VMMC |
| Staff can receive rewards for good performance | The staff at this facility can receive rewards for high or improved performance | HTC, PMTCT, VMMC |
| Funding linked to performance | Any part of the funding received by this facility is directly linked to the performance of the facility or its staff | PMTCT |
| Facility offers male reproductive health services | Facility offers detection and treatment of selected male sexual and reproductive health issues (e.g. infertility, erectile dysfunction, etc.) | VMMC |

**Abbreviations:** HTC: HIV testing and counselling; PMTCT: Prevention-of-mother-to-child-transmission;

VMMC: Voluntary medical male circumcision; PMAR: populations most-at-risk

### Measuring costs

Cost data were collected retrospectively by month for the entire calendar year 2011 or 2012 and for the month prior to data collection at the facility. To compute total costs of production, both the quantity and the unit price of each input were required. The ORPHEA instruments were designed to collect five categories of costs (Supplementary Exhibit A3). All donated inputs were valued at their unit costs determined by local price quotes. The study adopted an economic rather than financial costing perspective.

Supplementary Exhibit A3 ORPHEA cost categories listed with descriptions and examples

| Cost category | Description | Example |
| --- | --- | --- |
| Personnel | Staff time and costs by staff type and level for each working or volunteering individual in the facility’s HIV services production | Salaries, per diem, financial incentives for clinical and support staff |
| Recurrent supplies | Quantity and costs for individual or packaged supplies used in the delivery of HIV services | HIV test kits, male circumcision procedure kits, male condoms |
| Recurrent operating costs | Expenditures and types of utilities and goods that allow for the facility to continue operating | Utilities (gas, electric), rent, water |
| Capital | One-time purchases or acquisitions usually related to equipment, as well as any relevant maintenance or replacement costs | Equipment, vehicles, buildings |
| Other inputs | Any costs not mentioned above and relevant for maintaining administration, supervision, training, and other facility activities related to the facility in general and HIV services | Supervision, training |

We constructed six categories of staff according to the characteristics of the sample: medical staff, nurses, lab technicians, other health staff, support staff and midwives, as described in Supplementary Exhibit A4.

Supplementary Exhibit A4 ORPHEA staff categories (abbreviated)

| Categories | Definition |
| --- | --- |
| Medical staff | Clinical officers  Doctors  Medical officers  Surgeons |
| Nurses | Nurse practitioner  Nursing assistant  Nurse  Nurse, specialized |
| Lab technicians | Lab technician  Lab technologist |
| Other health staff | Counselor Health Worker (CHW)  Counselor  Health assistant  Peer educator  Volunteer  Pharmacist  Nutritionist |
| Support staff | Cleaning staff  Receptionist  Coordinator, HTC  Guard/watchman  Office assistant |
| Midwives | Midwives |

Staff costs comprised a large proportion of production costs,^[[6]](#footnote-6)^ and its measurement was not straightforward as health workers commonly work in more than one intervention. To this end, we employed time motion methods to measure staff time allocation by direct observation.^[[7]](#footnote-7),^^[[8]](#footnote-8)^

Time motion was employed at facilities where HIV prevention interventions were integrated (i.e., not stand alone) with other types of services. Using the staff roster at each facility, staff who satisfied the following inclusion criteria were eligible for time motion direct observation:

- worked at the facility at the time of the survey,
- directly provided services to clients,
- and split their time across more than one of the following services: HTC, PMTCT, VMMC, and other services.

From this list, data collectors observed up to six providers per facility. If six or fewer providers were eligible, all were sampled for time motion. If more than six providers were eligible, six were randomly selected for time motion. For each of the selected providers, data were collected on activities performed and the duration of time spent in each activity for approximately 3-4 continuous hours, outside of facility observation rooms. Observing providers from outside of their clinical space (e.g., having data collectors wait outside a nurse’s room) limited the Hawthorne effect, which predicts that study subjects would improve or modify their behavior when they know that they are being observed. It also respected ethical guidelines, as the providers’ work and time spent with patients were not interfered with by the study.

### Measuring outputs

Output data were also collected retrospectively by month for the entire calendar year 2011 or 2012 and for the month prior to data collection. For each intervention, we collected data on multiple outputs, corresponding as much as possible to the continuum in the cascade of services (see Supplementary Exhibit A5).

Supplementary Exhibit A5 Indicators along service cascades for HTC, PMTCT and VMMC

| **Intervention** | **Indicator along service cascade** |
| --- | --- |
| HTC | Clients tested |
|  | Clients tested and positive |
| PMTCT | Clients tested |
|  | Clients tested and positive |
|  | Clients on HAART |
| VMMC | VMMC procedures performed |

### Measuring process quality

We administered provider vignettes and client exit interviews for HTC, PMTCT and VMMC to assess service quality measures on provider competence and performance, respectively.^[[9]](#footnote-9)^

*Vignettes*

As a measure of provider competence, the vignette questionnaire assesses the extent to which health providers seem to follow existing guidelines on HIV prevention services. Health workers who had contact with HTC, PMTCT and VMMC clients at the facility were asked to respond in vignette interviews. Five vignettes were to be completed for each intervention – if fewer than five providers worked in one service, all providers were interviewed; if more than five providers worked in one service, five providers were randomly selected. Providers for each service were selected from the staff roster provided by the facility. The vignette for each intervention presented the respondent with a scenario capturing a hypothetical HTC, PMTCT or VMMC client, and each respondent was asked about how s/he would: take a medical history, perform a physical examination (if applicable), order appropriate laboratory or imaging tests, assess disease severity and functionality, and prescribe treatment (if applicable). The responses were then scored against national guidelines for each service.

*Exit interviews*

As a measure of provider performance, exit interviews assessed the process quality of services provided at the facility from the client’s perspective. The exit interview was directed at patients attending the facility for HTC, PMTCT or VMMC services. At each facility, data collection teams conducted up to five exit interviews for HTC and VMMC services each, and up to eight exit interviews for PMTCT services. For PMTCT the exit interviews captured different steps of the PMTCT cascade (e.g., antenatal care, maternity, postnatal care, nutrition counselling, etc.). The number of clients expected for each service was estimated based on the client register and/or the information obtained from health facility staff. If fewer than 10 clients were expected to receive care in the facility that day for each of the services, the enumerator interviewed the first five clients for HTC or VMMC and the first eight clients for PMTCT. When more than 10 clients were expected for a service, the enumerator randomly selected five HTC/VMMC clients or eight PMTCT patients. In addition to capturing the process quality of the services received (i.e., whether they received the most important elements of services and information of each service, according to the national guidelines), the exit interviews collected information on the reasons for visiting the facility, the type of health services received, access to health services, referrals from other facilities, and the content of the HIV prevention services received by the patient.

1. **Constructed Variables**

Since costs were specific to “sites” or “facilities,” not to interventions, we analyzed total production costs for each intervention across the sampled facilities based on the outputs reported for the period of interest.

- 1. **Personnel costs**

Typically, staff members performed a wide range of activities, and there was no *a priori* information about the relative distribution of their time across HIV and non-HIV activities. Therefore, calculating full-time equivalents (FTEs) was necessary to allocate staff costs to the specific HIV prevention interventions studied in the ORPHEA project.^[[10]](#footnote-10)^ We used an approach of allocation known as activity-based costing.^[[11]](#footnote-11)^

A FTE value of 1.0 represents full-time, whereas a FTE of 0.5 means that the staff in question works only half-time on HIV prevention and therefore only half of his or her salary should be included in the costs of producing prevention activities. Therefore, estimating staff costs of HIV prevention interventions involved two steps. First, time allocation factors for HTC, PMTCT and VMMC activities were estimated using data from the ORPHEA study’s time motion component. Second, the maximum number of hours that each staff member was expected to work during the period of observation (2011 or 2012) was used. Given that some staff members reported working more than 52 weeks per year, a ceiling at 52 was set for this variable. Costs of support staff (non-clinical) involved in the provision of HIV prevention services were prorated across interventions based on patient loads (service volume).

- 1. **Average costs and cost heterogeneity**

Annual costs for personnel, recurrent supplies, operating costs, and capital were estimated for each HIV prevention activity, whereas operating and capital costs, which were shared by all the clinics’ activities, were allocated based on the proportion of clients per intervention over the total number of outpatient clients recorded for the same year. In order to estimate the average annual cost per client, the total costs per intervention were divided by the total number of clients served by the same intervention. The average annual cost per client was plotted against the total number of clients per intervention in each facility and compared across facility types. All data on costs were converted from Kenya Shillings (KSH) to United States Dollar (USD) and annualized using 2011/2012 mid-year exchange rates (88.9 KSH = 1 USD). If current costs were provided for capital items, they were annualized and adjusted for inflation to match 2011.

- 1. **Determinants of efficiency**

Determinants of efficiency were extracted from the data to capture five dimensions of management quality: governance, accountability, supervision, monitoring, and incentives at each facility and related to specific HIV prevention services. Variables were either dichotomous (1=facility performs the activity; 0=facility did not perform the activity or respondent did not know) or count (the frequency of an event during year of observation (2011 or 2012)).

- 1. **Quality**

Considering the theoretical and empirical difficulties of evaluating service quality, we assessed process quality by measuring specific procedures and information received by the clients during their recent visit (exit interviews) or regularly provided by the staff (vignettes). Based on the data collected from provider vignettes and exit interviews, process quality scores for provider competence and performance, respectively, were constructed from the sum of a set of dummy variables for each intervention. These variables reflect general World Health Organization (WHO) and national recommendations for HTC, PMTCT and VMMC service delivery.

Individual (provider and patient) scores were given by the proportion of items the respondents correctly checked, so that 0=no quality at all, and 1=perfect quality. To compute facility-level measures we adopted two strategies. The performance scores were computed as the average of all the patient scores, while the competence score was computed as the maximum attained by any provider per facility, since we hypothesized that the knowledge of the best performing provider would trickle down into the rest of the facility.

- 1. **Imputation of missing data**

The following describes how incomplete or missing data were dealt with during the analysis stage.

- Personnel costs. We implemented an imputation process to fill missing values with a cascade algorithm, identifying the most immediate match given the covariates of provider type, contract type and gender.
- Recurrent operating costs (utilities). Data collected on utilities included electricity, water, grounds maintenance, building maintenance, telephone, diesel and oil/paraffin/kerosene. However, because the availability of these types of records was not always verifiable at the facility level and often difficult to obtain at other levels, the quality of these data was checked for potential strength for imputation. From this analysis, only missing values for electricity and water were subjected to imputation under a predictive mean matching using “mi impute pmm” syntax and procedures in Stata based on existing values for total number of beds, staff quantity, facility type, number of outpatient health clients, and total number of hours of operation.
- Cost of a single HIV test kit. For facilities without unit prices for HIV test kits, we replaced missing values with the unit price reported by a facility of the same type.
- Output indicators for service utilization. For facilities with 12 months of data, the values for all 12 months were added to yield annual outpatient client service volume; for facilities with six to 12 monthly observations, the monthly average for outpatient volume was computed with available data and imputed in the months with missing values. Facilities with fewer than six months of outpatient client data were dropped from the sample.
- Determinants of efficiency. Since most of the management responses were collected through interview with facility in-charges, it was expected that the respondent would have knowledge on the topic with regard to their facility and be able to provide an assured response.

1. **Theoretical and Empirical models**

We adopted an accounting identity approach^[[12]](#footnote-12)^ to characterize the relationship between the cost of a service and its determinants of a representative sample of $k$ facilities. If economies of scale are generated at the facility level only, we define total cost $TC$ for this sample of facilities as the sum of

$${tc}_{k}=A_{k}\times q_{k}^{\sigma}\times e^{\mathcal{Z}_{k}}$$

where $A_{k}=f(p_{k},\Gamma_{k})$, $\sigma$ is the elasticity of scale and a multiplicative vector of contextual and facility characteristics $\mathcal{Z}_{k}$ that shift the facilities’ technical efficiency. Average cost is defined as total cost divided by number of patients $q_{k}$, so the facility-specific average cost function $({atc}_{k})$ is defined as:

$${atc}_{k}=\frac{{tc}_{k}}{q_{k}}=\bar{A}\times q^{\sigma-1}\times e^{\mathcal{Z}_{k}}$$

Taking logs and setting $\beta=\sigma-1$, the model specification is:

$$\log{atc}_{k}=log\bar{A}+\beta log{(q}_{k})+\mathcal{Z}_{k}+\varepsilon_{k}$$

We can estimate this equation via OLS and test for the sign of the regression coefficients. If $\beta<0$, the data suggests the presence of economies of scale, $\beta=0$ denotes no economies of scale, and $\beta>0$ would be an indication of diseconomies of scale.

1. Health facility levels in the Kenya health system: Level 6=National referral hospital; Level 5=Provincial hospital; Level 4=District and Sub-district hospital; Level 3=Health centre; Level 2=Dispensary; and Level 1=Community-level interface. [↑](#footnote-ref-1)
2. Hsieh, Fushing Y., Daniel A. Bloch, and Michael D. Larsen. "A simple method of sample size calculation for linear and logistic regression." *Statistics in medicine*17.14 (1998): 1623-1634. [↑](#footnote-ref-2)
3. Marseille, Elliot, et al. "Assessing the efficiency of HIV prevention around the world: methods of the PANCEA project." *Health services research* 39.6p2 (2004): 1993-2012. [↑](#footnote-ref-3)
4. With the passing and adoption of the new constitution (August 27, 2010), Kenya has transited into a devolved system of governance in which the country is administratively divided into 47 counties (ROK 2010). [↑](#footnote-ref-4)
5. Note that the KAIS 2012 did not include North-Eastern, citing lack of sampling frame at the time of the survey. [↑](#footnote-ref-5)
6. Johns, Benjamin, and Tessa Tan Torres. "Costs of scaling up health interventions: a systematic review." *Health policy and planning* 20.1 (2005): 1-13. [↑](#footnote-ref-6)
7. Adam, Taghreed Mohamed Salama. "Sources of variability in costing methods: implications for transferability of cost-effectiveness results." (2006). [↑](#footnote-ref-7)
8. Bratt, John H., et al. "A comparison of four approaches for measuring clinician time use." *Health policy and planning* 14.4 (1999): 374-381. [↑](#footnote-ref-8)
9. Das, Jishnu, and Paul J. Gertler. "Variations in practice quality in five low-income countries: a conceptual overview." *Health Affairs* 26.3 (2007): w296-w309. [↑](#footnote-ref-9)
10. Tan, Siok Swan, et al. "Comparing methodologies for the cost estimation of hospital services." *The European Journal of Health Economics* 10.1 (2009): 39-45. [↑](#footnote-ref-10)
11. Baker, Judith J. "Activity-based costing for integrated delivery systems." *Journal of health care finance* 22.2 (1994): 57-61. [↑](#footnote-ref-11)
12. Meyer-Rath, Gesine, and Mead Over. "HIV treatment as prevention: modelling the cost of antiretroviral treatment—state of the art and future directions." *PLoS medicine* 9.7 (2012): e1001247. [↑](#footnote-ref-12)
